# The Influence of Person–Job Fit on Health Status and Depression Among Chinese Domestic Workers: Mediating Effect of the Employer–Employee Relationship

**DOI:** 10.3389/fpsyg.2022.782022

**Published:** 2022-06-23

**Authors:** Lijuan Chen, Maitixirepu Jilili, Ruolin Wang, Linping Liu, Anuo Yang

**Affiliations:** ^1^High-Quality Development Evaluation Institute, Nanjing University of Posts and Telecommunications, Nanjing, China; ^2^School of Social and Behavioral Sciences, Nanjing University, Nanjing, China; ^3^School of Economics and Management, Shihezi University, Shihezi, China

**Keywords:** person–job fit, mediating effect, health status, domestic workers, depressive symptoms

## Abstract

Domestic workers usually perform manual work in households. Unlike fixed work, their work tends to be individualized and atomized. Their person–job fit and relationship with employers might exert some influence on their health, both physical and psychological. This quantitative study explores the association between person–job fit, health status, and depressive symptoms among Chinese domestic workers by identifying the employer–employee relationship as a mediator. Data is collected from a survey of Chinese domestic workers conducted in four cities of Nanjing, Wuxi, Guangzhou, and Foshan (*N* = 1,003) in 2019. We test our theoretical model by conducting structural equation modeling. The results show that demand–ability fit is indirectly related to heath status and depressive symptoms *via* the employer–employee relationship. Need–supply fit is significantly associated with health status and depressive symptoms both directly (70% for health status and 72% for depressive symptoms, separately) and indirectly, *via* the mediating effect of the employer–employee relationship (30% for health and 28% for depressive symptoms, separately). Our findings suggest that appropriate policy and vocational training should be implemented to improve the health status of Chinese domestic workers.

## Introduction

With the implementation of China’s two-child policy, urbanization, and accelerated population aging, large- and medium-sized Chinese cities are experiencing a growing demand for domestic services. Some studies have explored the domestic service industry, including workplace health (Theodore et al., 2019) and the rights protection (Elias, 2010) and social identity (Vargas et al., 2020) of domestic workers. Domestic workers share numerous social characteristics. For example, most of them are female, relatively poorly educated, middle-aged, and economically disadvantaged. Previous studies have suggested that migrant workers, including domestic workers, are at high risk of experiencing poor health and depression due to their unfitness for work, long working hours, poor working conditions, and lack of health awareness (Li et al., 2007; Qiu et al., 2011; Lam and Johnston, 2015). Domestic workers are also highly likely to encounter occupational hazards, and their jobs often subject them to pain, discomfort, and poor working conditions (Zechter and Guidotti, 1987). In one study, Filipino domestic workers who work in Hong Kong scored low on health prevention. They experienced symptoms of mental distress such as waking up early, loneliness, anxiety, and difficulties falling asleep (Holroyd et al., 2001).

Scholars have studied migrant labor from countries such as the Philippines, India, and Indonesia (Amrith, 2021). However, most Chinese domestic workers are domestic floating population, many of whom are migrant workers. Compared to transnational labor, Chinese domestic workers experience fewer language and cultural barriers. They usually enter the industry through mutual introductions from townsmen groups and do not rely solely on domestic companies. In addition, the lack of self-organization also makes them vulnerable to occupational injuries (Jiang and Korczynski, 2021). This study explores the relationship between person–job fit and health status and depressive symptoms among domestic workers in China with specific reference to the mediating effect of the employer–employee relationship, and thus contributes to the literature on domestic workers’ health (both physical and psychological).

## Literature Review

### Person–Job Fit, Health Status, and Depression

Follow the definition proposed by Cable and DeRue (2002), Person–job (P–J) fit is the degree to which workers’ skills and job needs match. There are two facets of perceived P–J fit: demand–ability fit and need–supply fit. When an individual has the necessary knowledge, skills and abilities to meet the requirements of his job, the demand–ability fit can be achieved. When the job can meet the needs, preferences and desires of job seekers, a supply–demand fit can be achieved (Farzaneh et al., 2014). Therefore, P–J fit can be defined as the fit between the individual’s ability and work needs, or it can be defined as the fit between the individual’s needs and job attributes (Seikiguchi, 2004). For employees, person–job fit is important to job satisfaction (Tang et al., 2021), increasing job involvement (Ju et al., 2021), alleviating job stress, improving quality of work (Edwards, 1991). Employees with a greater demand–ability fit can finish their work more effectively, have less work pressure, and are more likely to win recognition and praise from their employers. Therefore, they are likely to report a better health status and less depression.

Lin et al. (2014) studied P–J fit and the influence of a sense of well-being on work outcomes. The results revealed a highly significant positive correlation between P–J fit and individual’s health. Apart from this, both P–J fit and health status were positively related to work performance. Choi et al. (2017) found a close link between P–J fit and employee health, because a good perceived P–J fit not only increases employees’ job satisfaction, but also alleviates their work-related stress and exhaustion. Rodrigues et al. (2020) found that work related demands, abilities, and needs could contribute to a process of adjustment, and consequently improve individuals’ psychological well-being.

### Employer–Employee Relationship, Health Status, and Depression

The employer–employee relationship plays a different role in the domestic service sector from that in a regular office context (Gurtoo, 2016), because most domestic service work is performed in employers’ “private spaces” and monitored by employers to some extent. Several studies have found a positive relationship between the employer–employee relationship, employees’ health status, and employees’ mental health problems (Shaw and Gupta, 2004; Qiu et al., 2011; Lam and Johnston, 2015). In many countries, women hold the majority of roles in the domestic service industry, including nursing, childcare, and housekeeping. This type of job is considered a low-status “female work,” it usually requires long hours of work and is vulnerable to abuse and exploitation (Gurtoo, 2016). Domestic workers tend to attach considerable importance to their interpersonal relationships at work and factors that make their jobs compatible with their responsibilities at home (Sorensen and Verbrugge, 1987). A cross-sectional study of working women in Malaysia provided evidence of an association between the employer–employee relationship and the health status of working women (Aazami et al., 2015). When the employer–employee relationship is positive, employees experience less work-related stress, anxiety, boredom, and exhaustion (Garabiles et al., 2019). In other words, a positive employer–employee relationship can enhance domestic workers’ commitment, motivation, and job satisfaction, which are highly correlated with their mental health (Furnham and Schaeffer, 1984; Park et al., 2011).

### P–J Fit, Employer–Employee Relationship, Health Status, and Depression

Previous studies have suggested that P–J fit can maximize work efficiency, increase job satisfaction, and enhance the employer–employee relationship (Babakus et al., 2010; Lin et al., 2014; Peng and Mao, 2015). Poor P–J matching is significantly associated with lower self-reported health status, and individuals tend to report more dissatisfaction, boredom, anxiety, depression, and physical symptoms (Warr and Ilke, 2012). P–J fit is related to improving overall job satisfaction, work adaptation, organizational commitment, and reducing worker’s willingness to resign (Seikiguchi, 2004). In this regard, a good P–J fit can strengthen the employer–employee relationship.

Research has found that the degree of personalization of relationships affects individuals’ perceptions of work relationships and workplace health. Whether the relationship with authority figures at work constitutes a key social background and further affects individuals’ perceptions of health and injury (Eakin and Maceachen, 2001). Edwards (1991), a forerunner in the development of the concept of perceived P–J fit, proposed that P–J is positively related to work performance, attendance, motivation and job satisfaction, and negatively related to work pressure. Research has also indicated that P–J fit is significantly associated with a series of work outcomes including work performance and job satisfaction (Caldwell and O’Reilly, 1990). Park et al. (2011) examined need–supply fit and demand–ability fit as two potential moderators of the relationship between core self-evaluation and health status and depression among Asian women. These two facets of P–J fit differed in the direction and magnitude of their relationships with indicators of health status. Need–supply fit was significantly related only to happiness, not to depression, but demand–ability fit was significantly related to depression. Anjara et al. (2017) examined the factors influencing the health and quality of life among female migrant domestic workers in Singapore. The results indicated that even though the overall quality of life of these domestic workers was found to be good and they were sufficiently satisfied with their own health status, more than half of them still felt stressed. Bagley et al. (1997) conducted a study focused on the mental health status of Filipino domestic helpers in Hong Kong and revealed correlations between the factors that they found to be stressful and high levels of mental health impairment.

### Research Gaps

Previous research in this field mainly focused on the positive effects of P–J fit on employees’ innovative behavior, work attitude, health and job satisfaction, and employer–employee relationship (Lin et al., 2014; Choi et al., 2017; Cai et al., 2018; Kim et al., 2018; Lim et al., 2019; Tang et al., 2021). Although the impact of P–J fit on the physical and mental health of domestic workers has attracted attention from scholars worldwide, studies in the Chinese context are lacking. In addition, prior studies have not explored the indirect effects of P–J fit on health and depressive symptoms *via* the employer–employee relationship among Chinese domestic workers.

### Current Study

**Figure 1:**
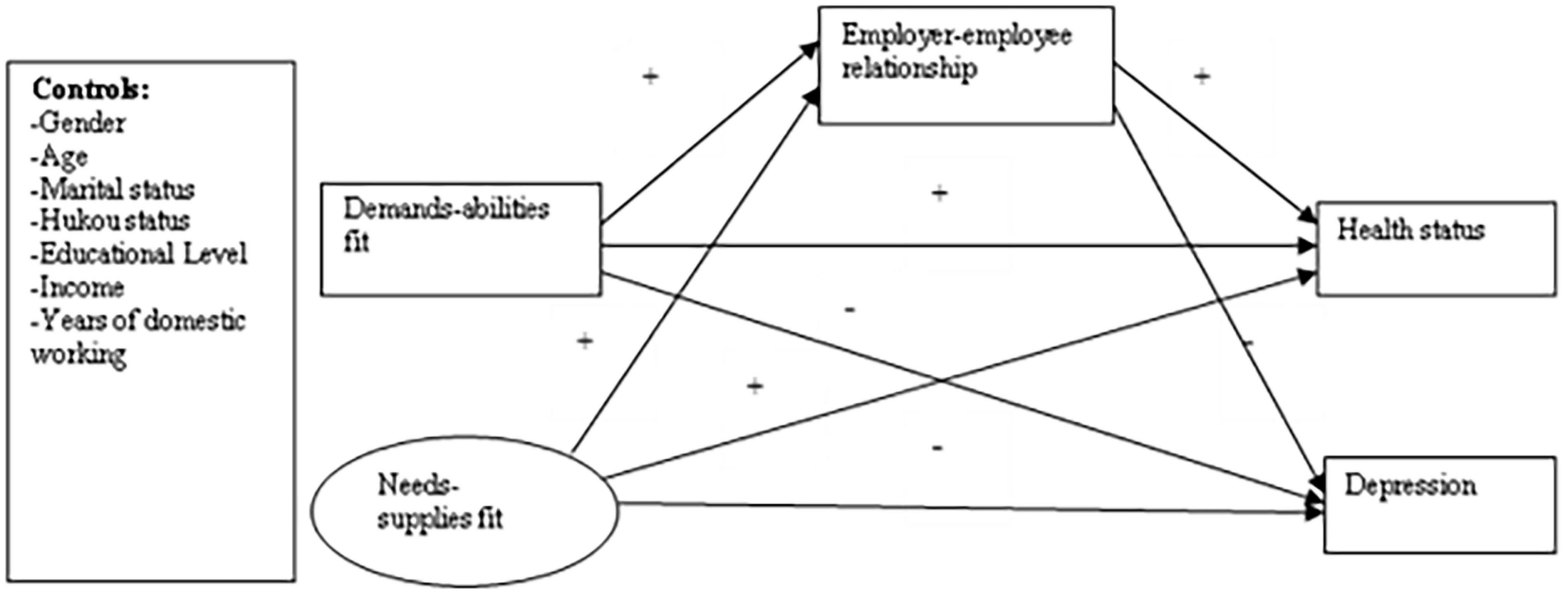
Hypothesized process model.

In this research, we mainly focus on two health indicators of Chinese domestic workers: self-reported health status and depression. Using survey data, we explored the association between P–J fit, health status, and depressive symptoms, and introduced the mediating role of the employer–employee relationship in this relationship. Based the existing literature, we constructed the conceptual model (see Figure 1) and proposed the following hypotheses:

*H1*: A greater P–J fit is associated with a better self-reported health status among Chinese domestic workers.

*H2*: A greater P–J fit is associated with lower levels of depression among Chinese domestic workers.

*H3*: A greater P–J fit is associated with a better employer–employee relationship among Chinese domestic workers, thereby improving the workers’ health status.

*H4*: A greater P–J fit is associated with a better employer–employee relationship among Chinese domestic workers, thereby alleviating the workers’ depression symptoms.

## Materials and Methods

### Data

Data were obtained from a survey of domestic workers conducted by our research group at Nanjing University in 2019. This survey was designed to collect sociodemographic, economic, and domestic work information among Chinese domestic workers and administered across four Chinese cities: Nanjing, Wuxi, Guangzhou, and Foshan. The former two cities are in the Yangtze River Delta, while the latter two are in the Pearl River Delta. Both of these regions are densely populated and economically developed. Given the difficulty of obtaining sampling frames, this survey followed a respondent driven sampling (RDS) approach, which was introduced by Heckathorn in the mid-1990s (Heckathorn, 1997). RDS has proven to be particularly suitable for sampling hidden groups, allowing inferences to be made about groups for which traditional sampling methods are not feasible or practical (Salganik and Heckathorn, 2004; Volz and Heckathorn, 2008; Gile and Handcock, 2010; Treiman et al., 2012). Using RDS diagnostic tools and geographical data analysis (Schonlau and Liebau, 2012; Gile and Handcock, 2015; Kim et al., 2020), survey data evaluations revealed that key demographic information such as education and age showed consistency within the same city, which indicated that the samples obtained can be representative. The sampling and recruitment procedures were determined after obtaining ethical approval from the Central Ethical Review Committee of the Research Council of Nanjing University.

### Participants

In the process of RDS, a sample is collected based on respondents’ recommendations. The first respondent to be interviewed is called the “seed” respondent. During each interview, the respondent is encouraged to invite other suitable participants, and this process continues until the sample achieves equilibrium. We trained interviewers to administer the questionnaire survey and guide the seed and other respondents to invite other domestic workers to participate in the survey. In total, 1,007 domestic workers completed the questionnaire. After deleting responses with missing data, our final sample for analysis consisted of 1,003 domestic workers. The average age of the respondents was 50.13 years (SD = 6.77), and 983 were female (98.01%), while 20 were male (1.99%). Table 1 presented the descriptive characteristics of the sample.

**Table 1:** Descriptive characteristics (*N* = 1,003).

	Frequency (*N*)/Mean	Percentage (%)/Standard Deviation
Health status	Mean = 4.28	SD = 0.78
Depression	Mean = 4.29	SD = 4.36
Relationship with employer	Mean = 4.29	SD = 0.70
*Person–job fit*		
Demands-abilities fit	Mean = 4.14	SD = 0.84
Needs-supplies fit	Mean = 0.00	SD = 0.84
Age	Mean = 50.13	SD = 6.77
*Gender*		
Female	*N* = 983	98.01%
Male	*N* = 20	1.99%
Hukou status		
Rural	*N* = 748	74.58%
Urban	*N* = 255	25.42%
*Education level*		
Illiterate	*N* = 60	5.98%
Primary school	*N* = 296	29.51%
Junior high school	*N* = 494	49.25%
Senior high school/Junior college	*N* = 137	13.66%
College and above	*N* = 16	1.60%
*Marital status*		
Unmarried	*N* = 115	11.47%
Married	*N* = 888	88.53%
Income	Mean = 10.48	SD = 1.58
Years of domestic working	Mean = 8.48	SD = 6.90

### Measurement

#### Health Status and Depression

##### Health Status

As mentioned earlier, some studies have shown that self-reported health status is a good indicator of health (Idler and Benyamini, 1997; Manderbacka et al., 2003). In this domestic worker survey, respondents were asked to report their health status. The options were coded on a 5-point scale, from 1 (very unhealthy) to 5 (very healthy).

##### Depression

The Center for Epidemiologic Studies–Depression (CES-D) scale (Radloff, 1977) is specially designed to evaluate the frequency of current depressive symptoms, focusing on depressive emotion or mood. Our research used a shortened version of the scale, comprising only 10 items (CES-D-10, Andresen et al., 1994). Both the reliability and the sensitivity of the CES-D-10 are comparable to the original CES-D (Irwin et al., 1999). It comprises 10 items, including “I was bothered by things that usually do not bother me,” “I had trouble keeping my mind on what I was doing,” and “I felt depressed.” Responses were coded as 0 (rarely or none of the time), 1 (some or a little of the time), 2 (occasionally or for a moderate amount of time), or 3 (most or all of the time). The scores were summed (range: 0–26). The higher the aggregate scores mean the more severe the symptoms of depression.

#### P–J Fit

P–J fit reflects the matching of individual’s knowledge, skills and abilities with job needs (Carless, 2005). Prior researchers developed perceptions of fit in three dimensions: person–organization fit, need–supply fit, and demand–ability fit (Cable and Derue, 2002). As domestic workers usually work in their employers’ homes and their work environments often lack organization, we used only two of these dimensions as indicators of P–J fit, namely demand–ability fit and need–supply fit. P–J fit is achieved when employees have the technical ability to meet the job needs or the job can meet the employee’s needs (Edwards and Van Harrison, 1993; Kristofbrown, 2000). The item “My knowledge, technique, and abilities are a good fit with the requirements of my job” was employed to measure demand–ability fit. Need–supply fit was measured using a three-item scale, based on Cable and DeRue (2002). The questions were “To what extent does your current job meet your needs?,” “To what extent does your current job suit you?,” and “To what extent is your current job what you want to do?” All options were given on a 5-point scale ranging from 1 (very little) to 5 (a large extent). As the measure of demand–ability fit comprised just one question, while the measure of need–supply did not, we performed confirmatory factor analysis and extracted a single factor as our measurement. Higher scores suggested a better demand–ability fit and need–supply fit.

#### Employer–Employee Relationship

The employer–employee relationship was measured using the question “To what extent you are satisfied with your relationship with your employer?” The options used a five-point scoring method, ranging from 1 “very dissatisfied” to 5 “very satisfied.”

#### Socio-Demographic Variables

Our structural models controlled demographic and socio-economic factors, including age, gender (code “0” for female and “1” for male), *hukou* status (code “0” for rural and “1” for urban), education level (code “1” for illiterate, code “2” for primary school, code “3” for junior high school, code “4” for senior high school/junior college, and code “5” for college and above), marital status (code “0” for unmarried and “1” for married), ethnicity (code “0” for minority group and “1” for Han), and income (logged), and years of domestic work.

### Statistical Analysis

Structural equation modeling was used to test our conceptual model. Our analyses consisted of two stages (Anderson and Gerbing, 1988). First, measurement model *via* confirmatory factor analysis to extract a need supply fit factor was tested. Second, we constructed a structural model to estimate the mediating effects and determine whether the hypothesized model was empirically supported. The *χ*^2^ values is inevitably affected by the sample size and constant degrees of freedom, which will make plausible models have the risk of being rejected due to the significant *χ*^2^, even if the contradiction between the sample and the model’s implicit covariance matrix is actually unrelated (Tanguma, 2001). We used the following fit indices to evaluate the model’s fitness: the normed fit index (NFI), comparative fit index (CFI), and root mean square error of approximation (RMSEA). According to Kaplan (2000), the rule of thumb for the NFI is that values greater than 0.95 indicate a good goodness of fit relative to the baseline model. NFI values greater than 0.90 but less than 0.95 is generally considered acceptable goodness of fit (Marsh and Grayson, 1995; Schumacker and Lomax, 1996). If the value of the CFI is greater than 0.90, the model has a good fit, and beyond 0.95, the model fit is very good (Bentler, 1990). RMSEA value less than or equal to 0.05 indicates a close goodness of fit, and a value between 0.05 and 0.08 indicates that a sufficient goodness of fit (Steiger, 1990; Browne and Cudeck, 1992; Kline, 2005).

Given that the survey used a self-reported questionnaire and all the variables measures were collected from respondents at the same time, we conducted Harman’s one-factor test and confirmatory factor analysis to assess potential problems with common method variance statistically (Harman, 1967; Podsakoff, 2003). An exploratory factor analysis (EFA) with all study variables produces seven factors with eigenvalues greater than 1 and the first factor accounts for 26% (less than 50%) of the variance among variables. The poor goodness of fit of confirmatory factor analysis also indicated that there were not severely common method variance (NFI = 0.605, CFI = 0.619, RMSEA = 0.112). All of the analyses were conducted using Amos (version 21). Table 1 showed the descriptive characteristics calculated from the total sample.

## Results

### Test of the Measurement Model

Kaiser-Mayer-Olkin (KMO) test and Bartlett test of sphericity were employed to measure the sampling adequacy and the correlations between the variables, respectively. The KMO was 0.653, which indicates a mediocre degree of sampling adequacy, and the chi-square value in the Bartlett test was 830.338 (*p* < 0.001), indicating that the data were suitable for factor analysis. The freedom of the measurement model was 0, making it an exact recognition model in which all of the parameters had only unique solutions. The analysis also indicated that the demand–ability fit measure was reliable (Cronbach’s *α* = 0.752, where values greater than 0.70 signify an acceptable fit; Nunnally, 1978). Table 2 presented the standardized factor loadings of the observable variables. Based on the research of Agnew (1991), all of the factor loadings, which ranged from 0.566 to 0.890, were acceptable (above 0.3).

**Table 2:** Standardized factor loadings of the observable variables.

Latent construct	Observed variable	Factor loading
*Person–job fit*		
Needs-supplies fit	To what extent your current job meets your needs	0.566
	To what extent your current job suits you	0.890
	To what extent your current job is what you want to do	0.716

### Test of the Structural Model

**Figure 2:**
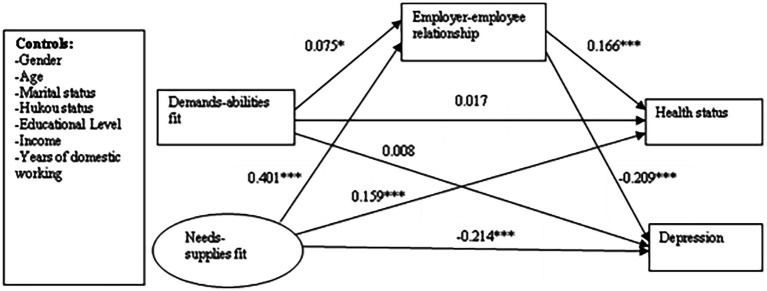
Standardized solutions for the structural model of person–job fit, health status and depression of the domestic workers. ^*^p < 0.05 and ^***^p < 0.001.

As the RMSEA provides a robust measure of the quality of the tested model, it is commonly accepted as one of the best fit indices available (Maccallum and Austin, 2000). Combined with the value of the NFI and CFI indicators, our final tested structural model showed a good fit with the data (NFI = 0.957, CFI = 0.980, RMSEA = 0.028). Figure 2 presents the standardized estimates for the structural model combining the P–J fit, health status, and depression of domestic workers.

The direct effects of demand–ability fit on self-reported health status and depressive symptoms did not reach significance, but the indirect effects *via* the employer–employee relationship were statistically significant. Both the direct and indirect effects of need–supply fit on health status and depression were statistically significant. Therefore, a greater P–J fit is related to a better health status and reduced feelings of depression among Chinese domestic workers.

Demand–ability fit and need–supply fit were each significantly positively correlated with a better employer–employee relationship (*β* = 0.075, *p* < 0.05 for demand–ability fit and *β* = 0.401, *p* < 0.001 for need–supply fit). A better employer–employee relationship may improve self-reported health status (*β* = 0.166, *p* < 0.001) and alleviate depression symptoms (*β* = −0.209, *p* < 0.001). The results suggest that the employer–employee relationship is a significant mediator between the impact of P–J fit on health status and depressive symptoms among Chinese domestic workers.

Further, we decomposed the standardized total effects, direct effects, and indirect effects of P–J fit on health status and depression. The results are presented in Table 3. The total effect of demand–ability fit on health status was 0.030 and that on depression was −0.007, combining the direct effect (0.017 for health status and 0.008 for depression) and the indirect effect through the employer–employee relationship (0.012 for health status, −0.016 for depression). The total effect of need–supply fit on health status was 0.226 and that on depression was −0.297, combining the direct effect (0.159 for health status, −0.214 for depression) and the indirect effect through the employer–employee relationship (0.067 for health status, −0.084 for depression). We further used the bootstrapping method to test the indirect effects. For demands-abilities fit, the confidence intervals of indirect effect ranged from 0.060 to 0.133 on health status, and ranged from −0.952 to −0.481 on depression. For needs-supplies fit, the confidence intervals of indirect effect ranged from 0.003 to 0.023 on health status, and ranged from −0.157 to −0.019 on depression. All of the confidence intervals for the indirect effects on health status and depression did not include 0, indicating that the mediating effects were statistically significant.

**Table 3:** Standardized total effects, direct effects, and indirect effects of person–job fit on health status and depression.

			Direct effects	Indirect effects	Total effects
Demands-abilities fit	→	Health status	0.017	0.012	0.030
Needs-supplies fit	→	Health status	0.159	0.067	0.226
Demands-abilities fit	→	Depression	0.008	−0.016	−0.007
Needs-supplies fit	→	Depression	−0.214	−0.084	−0.297

## Discussion

Prior studies have shown that P–J fit has an important impact on health and depression. Further, P–J fit may be particularly important to domestic workers, whose poor working conditions, long working hours, and relative lack of education make them vulnerable to poor health and depression. Accordingly, we hypothesized that a good P–J fit leads to less symptoms of depression and better health status among Chinese domestic workers, and these effects arise partly through the interaction between P–J fit and the employer–employee relationship.

Using data from four cities in China, we found that a better P–J fit was related to an improved health status and a reduced frequency of depression, which supported H1 and H2 of our study. We also found that these associations were mediated by the employer–employee relationship, which supported H3 and partially supported H4. In our model, the employer–employee relationship accounted for over 28% of the effect of need–supply fit on health status and depressive symptoms, and the effects of demand–ability fit on health status and depressive symptoms were mostly indirect, *via* the employer–employee relationship.

This study contributes to literature among domestic workers in China. Prior studies have paid less attention to Chinese domestic workers. As workers in a disadvantaged position in social welfare and social security, their physical and mental health should receive more research concerns. Investigating the mediating effects of employer–employee relationship is another asset of this study. Given that the main workplaces of domestic workers are the employers’ houses, their relationships with the employers have a significant impact on their physical and mental health and mediate the associations with P–J fit and health and depressive symptoms.

Our findings have several important practical implications. They suggest that P–J fit has a positive influence on employee health status and depression among Chinese domestic workers. Thus, to enhance domestic workers’ health, both employers and employees should attach great importance to perceived fit to specific jobs. The relationships of demands-abilities fit and health outcomes are completely mediated by the employer–employee relationship, suggesting that vocational training should not only pay attention to labor skills, but also should provide guidance on how to build better employee relationships. Establishing a strong, positive, and harmonious employer–employee relationship to improve workers’ physical and mental health. Needs-supplies fit has both direct and indirect associations with health outcomes of domestic workers which might call for more attention to individual needs and individualized support in social work practice. Establishing self-organization of domestic workers can improve the ability of negotiating with employers and domestic companies, better meet the needs of individual rights, and can also be used as an effective intervention. Generally, Chinese domestic workers are prone to occupational stress and health related problems because of the characteristics of their work. This study offers highly significant insights into the self-rated health status and depression of Chinese domestic workers from the perspective of P–J fit perception, especially in presenting the employer–employee relationship as a mediator.

### Limitations

There are some limitations of our study that deserve discussion and point to directions of future research. The first limitation relates to the measure of demand–ability fit used in the study, which was adapted from Cable and DeRue (2002). The original three items measuring demand–ability fit, “knowledge,” “technique,” and “abilities,” were combined in this study, which may have led to measurement error. Second, person-organization fit was not included in the analytical framework. Though the main influence on domestic workers came from their employers, the potential impact of person-organization fit on P–J fit and employment relationships might cause estimate bias. Third, we focused on the correlations rather than the causal relationships between P–J fit, employer–employee relationship, health status, and depression. As the research design and cross-sectional methodology were restricted in their ability to construct causal relationships, further longitudinal analysis could be performed in the future.

### Conclusion

This study highlights the direct effect of P–J fit on health status and depression among Chinese domestic workers and the indirect effect of these associations *via* employer–employee relationship. The results reveal that a good P–J fit, including demand–ability fit and need–supply fit, improves workers’ health status and reduces their symptoms of depression. These associations can be interpreted partly in terms of the mediating effect of the employer–employee relationship. Considering that Chinese domestic workers are in a disadvantaged work condition, these results can provide significant implications for improving their physical and mental health.

## Data Availability Statement

The raw data supporting the conclusions of this article will be made available by the authors, without undue reservation.

## Ethics Statement

The studies involving human participants were reviewed and approved by Ethics Committee of the Department of Sociology, Nanjing University. The patients/participants provided their written informed consent to participate in this study.

## Author Contributions

LL, MJ, and LC contributed to conception and design of the study. LC and RW organized the database and performed the statistical analysis. AY revised the first draft of the manuscript. All authors contributed to the article and approved the submitted version.

## Funding

This research was supported by the Key Project of National Social Science Fund of China (18ASH007) and Jiangsu Provincial Social Science Planning Project (21WTA-018).

## Conflict of Interest

The authors declare that the research was conducted in the absence of any commercial or financial relationships that could be construed as a potential conflict of interest.

## Publisher’s Note

All claims expressed in this article are solely those of the authors and do not necessarily represent those of their affiliated organizations, or those of the publisher, the editors and the reviewers. Any product that may be evaluated in this article, or claim that may be made by its manufacturer, is not guaranteed or endorsed by the publisher.
